# EVIDENCE OF THE INFLUENCE OF GLUCAGON-LIKE PEPTIDE ANALOGS IN INFLAMMATORY BOWEL DISEASE: A SCOPING REVIEW

**DOI:** 10.1590/S0004-2803.24612026-028

**Published:** 2026-08-14

**Authors:** Lucas Dalvi Armond REZENDE, Camila ADOUR, Gabriel Confalonieri BERTOLDI, Marco Antônio Oliveira BRITO, Flávia Lúcia CONCEIÇÃO, Mariana Poltronieri PACHECO

**Affiliations:** 1 Centro Universitário Salesiano - UniSales, Departamento de Enfermagem, Vitória, ES, Brasil.; 2 Universidade Federal do Rio de Janeiro, Departamento de Endocrinologia, Rio de Janeiro, RJ, Brasil.; 3 Hospital Federal do Andaraí, Departamento de Gastroenterologia, Rio de Janeiro, RJ, Brasil.; 4 Universidade Federal do Rio de Janeiro, Departamento de Clínica Médica, Rio de Janeiro, RJ, Brasil.; 5 Escola Superior de Ciências da Santa Casa de Misericórdia de Vitória - Emescam, Departamento de Medicina, Vitória, ES, Brasil.; 6 Hospital Santa Casa de Misericórdia de Vitória (HSCMV), Departamento de Gastroenterologia e Hepatologia, Vitória, ES, Brasil.

**Keywords:** Glucagon-like peptides, crohn disease, ulcerative colitis, Peptídeos semelhantes ao glucagon, doença de Crohn, colite ulcerative

## Abstract

**Background:**

Inflammatory bowel disease is a chronic, immune-mediated condition characterized by relapsing intestinal inflammation and progressive tissue damage. Despite advances in biological and small-molecule therapies, a considerable proportion of patients experience suboptimal response, treatment intolerance, or persistent disease activity. In parallel, the growing prevalence of obesity and type 2 diabetes among individuals with inflammatory bowel disease has raised interest in therapeutic agents capable of modulating both metabolic dysfunction and intestinal inflammation. Glucagon-like peptide-1 and glucagon-like peptide-2 receptor agonists have demonstrated anti-inflammatory and intestinotrophic properties in experimental and clinical settings, suggesting a potential role in this population.

**Objective:**

To systematically map and synthesize current scientific evidence regarding the effects of glucagon-like peptide-1 and glucagon-like peptide-2 receptor agonists in patients with inflammatory bowel disease.

**Methods:**

A scoping review was conducted following established methodological frameworks for evidence synthesis. A comprehensive search of electronic databases was performed to identify pre-clinical, observational, and interventional studies evaluating the effects of glucagon-like peptide-1 and glucagon-like peptide-2 receptor agonists in inflammatory bowel disease. Studies were screened according to predefined eligibility criteria. Data extraction focused on mechanisms of action, inflammatory markers, clinical outcomes, safety profile, and therapeutic implications.

**Results:**

Pre-clinical studies consistently demonstrated that glucagon-like peptide-1 receptor agonists reduce intestinal inflammation through modulation of immune signaling pathways, suppression of pro-inflammatory cytokines, and enhancement of epithelial barrier integrity. Observational human studies suggest potential clinical benefits, including reduction in disease activity and decreased need for corticosteroid escalation in selected patients. Glucagon-like peptide-2 analogs showed pronounced effects on mucosal regeneration, epithelial proliferation, and improved nutrient absorption, particularly in patients with Crohn’s disease and intestinal failure. Overall, the available evidence indicates promising metabolic and intestinal benefits; however, most studies were limited by small sample sizes and heterogeneous designs.

**Conclusion:**

Glucagon-like peptide-based therapies represent a promising adjunctive strategy in inflammatory bowel disease due to their anti-inflammatory and regenerative properties. Nevertheless, high-quality randomized controlled trials focusing on diseasespecific clinical, endoscopic, and safety outcomes are necessary to establish their efficacy and therapeutic positioning in routine care.

## HIGHLIGHTS

• Scoping review investigating the role of GLP-1 and GLP-2 receptor agonists in inflammatory bowel disease (IBD).

• Comprehensive analysis of pre-clinical, observational, and clinical studies conducted according to PRISMA-ScR methodology.

• It suggests that GLP-1 receptor agonists demonstrate anti-inflammatory and immunomodulatory effects, while GLP-2 analogs promote mucosal regeneration and intestinal activity.

• Evidence suggests potential dual metabolic and intestinal benefits; however, randomized controlled trials are still required to confirm efficacy and safety.

• Study conducted by a multidisciplinary team including a Clinical Nurse Specialist, Gastroenterologist, Endocrinologist, and General Practitioner.

## RESUMO

**Contexto:**

A doença inflamatória intestinal é uma condição crônica, imunomediada, caracterizada por inflamação intestinal recorrente e dano tecidual progressivo. Apesar dos avanços nas terapias biológicas e em pequenas moléculas, parcela significativa dos pacientes apresenta resposta subótima, intolerância terapêutica ou atividade inflamatória persistente. Paralelamente, o aumento da prevalência de obesidade e diabetes tipo 2 entre indivíduos com doença inflamatória intestinal tem ampliado o interesse por terapias capazes de modular simultaneamente disfunções metabólicas e inflamação intestinal. Agonistas dos receptores do peptídeo semelhante ao glucagon 1 e 2 têm demonstrado propriedades anti-inflamatórias e intestinotróficas em modelos experimentais e estudos clínicos, sugerindo potencial aplicabilidade nessa população.

**Objetivo:**

Mapear e sintetizar sistematicamente as evidências científicas atuais acerca dos efeitos dos agonistas dos receptores do peptídeo semelhante ao glucagon 1 e 2 em pacientes com doença inflamatória intestinal.

**Métodos:**

Realizou-se uma revisão de escopo seguindo referenciais metodológicos consolidados para síntese de evidências. Foi conduzida busca abrangente em bases de dados eletrônicas para identificação de estudos pré-clínicos, observacionais e intervencionais que avaliaram os efeitos desses agonistas na doença inflamatória intestinal. Os estudos foram selecionados conforme critérios de elegibilidade previamente definidos. A extração de dados contemplou mecanismos de ação, marcadores inflamatórios, desfechos clínicos, perfil de segurança e implicações terapêuticas.

**Resultados:**

Estudos pré-clínicos demonstraram consistentemente que agonistas do receptor do peptídeo semelhante ao glucagon 1 reduzem a inflamação intestinal por meio da modulação de vias imunológicas, supressão de citocinas pró-inflamatórias e fortalecimento da integridade da barreira epitelial. Estudos observacionais em humanos sugerem potenciais benefícios clínicos, incluindo redução da atividade da doença e menor necessidade de escalonamento de corticosteroides em populações selecionadas. Análogos do peptídeo semelhante ao glucagon 2 apresentaram efeitos relevantes na regeneração mucosa, proliferação epitelial e melhora da absorção de nutrientes, especialmente em pacientes com doença de Crohn e insuficiência intestinal. Entretanto, a maioria dos estudos apresentou amostras reduzidas e delineamentos heterogêneos.

**Conclusão:**

Terapias baseadas em peptídeos semelhantes ao glucagon configuram estratégia promissora adjuvante na doença inflamatória intestinal, devido às suas propriedades anti-inflamatórias e regenerativas. Contudo, ensaios clínicos randomizados de alta qualidade são necessários para estabelecer sua eficácia, segurança e posicionamento terapêutico na prática clínica.

## INTRODUCTION

Inflammatory bowel diseases (IBD), such as Crohn‘s disease (CD) and ulcerative colitis (UC), are chronic inflammatory bowel conditions with a complex and poorly understood pathogenesis, involving genetic, immunological, and environmental factors^1^. CD inflammation involves the entire intestinal wall and can occur anywhere in the gastrointestinal tract, while in UC, only the colonic mucosa is directly affected^2^.

In recent decades, the epidemiology of IBD has shown significant changes worldwide. Traditionally more prevalent in developed countries in North America and Europe, these diseases have shown a significant increase in emerging regions such as Latin America, Asia, and Africa. This phenomenon largely reflects the Westernization of lifestyle, with changes in dietary habits, greater exposure to antibiotics, urbanization, and reduced contact with environmental microorganisms. It is estimated that more than 7 million people currently live with IBD worldwide, and the trend is for continued growth, especially in low- and middle-income countries, where diagnosis and access to advanced therapies still represent significant challenges^3^.

Concurrently, there has been a global rise in obesity and type 2 diabetes mellitus, conditions that share inflammatory and metabolic pathophysiological pathways with IBD. Emerging evidence indicates that patients with IBD and obesity tend to have a more severe clinical course, a higher risk of hospitalization and surgical intervention, and a reduced response to conventional biological therapies. Furthermore, approximately 30-50% of patients fail to respond adequately to available treatments or experience secondary loss of response over time, underscoring the need for therapeutic strategies that integrate intestinal inflammation control with the management of associated metabolic dysfunction. This scenario supports the growing recognition of the interplay among immunity, gut microbiota, and metabolism as a central axis for innovative therapeutic approaches. The prevalence of multimorbidity is increasing, and patients with IBD may present with coexisting conditions, such as diabetes. In the context of concomitant medication use, treatment for one condition may be contraindicated for another; conversely, there are situations in which therapy for one chronic disease may positively influence a concurrent condition^4^.

Glucagon-like peptide-based medications, also known as incretin-based therapies, are effective in the management of type 2 diabetes due to their glucose-lowering mechanisms^5^ ^,^ ^6^. Glucagon-like peptide-1 exerts important metabolic functions, including stimulation of insulin secretion, inhibition of glucagon release, and anorexigenic effects, thereby contributing to glycemic and weight control. Additionally, it demonstrates direct anti-inflammatory properties, including inhibition of pro-inflammatory pathways such as nuclear factor kappa B, reduction of cytokines such as tumor necrosis factor alpha and interleukin 6, and modulation of immune cells toward a more tolerogenic profile. Glucagon-like peptide-2 acts as an intestinal growth factor, stimulating crypt cell proliferation, reducing apoptosis, expanding mucosal surface area, enhancing nutrient absorption, and strengthening the intestinal barrier. These mechanisms support the growing interest in investigating the efficacy and safety of glucagon-like peptide-1 and glucagon-like peptide-2 receptor agonists as adjunctive therapies in the treatment of Crohn’s disease and ulcerative colitis^7^ ^,^ ^8^.

Given the clinical and epidemiological relevance of IBD, as well as the potential influence of glucagon-like peptides on inflammatory modulation and disease management, this study aimed to synthesize the available evidence regarding the potential effects of glucagon-like peptide analogs in inflammatory bowel diseases.

## METHODS

This is a scoping review that consists of synthesizing research evidence, with the aim of mapping the existing literature on a given subject. This scoping review had its research protocol registered in the Open Science Framework, under the Doi: 10.17605/OSF.IO/SDQCH.

The construction process was carried out in two complementary stages: (1) identification of peer-reviewed literature in electronic databases; and (2) synthesis and organization of the retrieved information, resulting in the synthesis of the scoping review.

To construct the study, the step-by-step guide proposed by JBI was used, with a methodological path organized into 11 sequential steps: 1 - Preliminary literature review; 2 - Construction of the research protocol; 3 - Collection of information from the authors of the sample studies; 4 - Development of the title, objective, and research question; 5 - Inclusion criteria; 6 - Research strategy; 7 - Data collection and background; 8 - Theoretical discussion; 9 - Extraction of results; 10 - Analysis of evidence; and 11 - Presentation of results^9^.

As this is a review study, there was no need for approval from the Research Ethics Committee. This review was developed based on the recommendations of the international guide Preferred Reporting Items for Systematic Reviews and Meta-Analyses extension for Scoping Reviews (PRISMA-ScR) and the review method proposed by the Joanna Briggs Institute (JBI) for scoping reviews^9^.

To guide the formulation of the guiding question, the PCC mnemonic strategy (population/problem, concept, context) was adopted. Therefore, the following were considered: problem - influence of GLP on inflammatory bowel diseases; concept of interest - inflammatory bowel disease; and research context - scientific literature. The research question, outlined under the acronym PCC, was: “What is the evidence regarding the influence and role of GLP in inflammatory bowel disease?”.

### Study eligibility

Primary quantitative or qualitative studies were included. Technical and governmental documents, as well as theses and dissertations, were also considered, excluding preprints, and with a publication time limit of 10 years. Regarding the selected languages, studies in English, Portuguese, Spanish, and French were included.

Studies that did not directly address the influence, pathophysiological role, or clinical effects of glucagon-like peptides (GLP-1, GLP-2, or their analogues) in inflammatory bowel diseases were excluded from the present review. Articles whose primary focus was limited to the management of diabetes mellitus, obesity, or other metabolic conditions, without explicit analysis or clinical applicability to IBD populations, were also excluded, including studies that did not discuss applicability in non-diabetic populations.

### Data collection and extraction from studies

The search was conducted in May 2025 in the following databases: Medical Literature Analysis and Retrieval System Online - MEDLINE/PubMed, Cochrane Library, Nursing Database (BDENF), Latin American and Caribbean Literature in Health Sciences (LILACS), Spanish Bibliographic Index in Health Sciences (IBECS), National Center for Medical Sciences Information of Cuba (CUMED), Western Pacific Index Medicus (WPRIM), Argentine National Bibliography in Health Sciences (BINACIS), and Embase. For the search in the aforementioned databases, the following Health Science Descriptors/Medical Subject Headings were chosen: “Glucagon-Like Peptide 1”, “Glucagon-Like Peptide 2”, “Glucagon-Like Peptides”, “Glucagon-Like Peptide Receptors”, “Granulomatous colitis” OR “Regional Enteritis”, “Crohn‘s Disease”, “Colitis”, “Hemorrhagic Colitis”, “Ulcerative Colitis”. For the research, the descriptors were separated by Boolean operators, thus, the search was summarized as: (“Glucagon-Like Peptide 1” OR “Glucagon-Like Peptide 2” OR “Glucagon-Like Pep­tides” OR “Glucagon-Like Peptide Receptors”) AND (“Granulomatous colitis” OR “Regional Enteritis” OR “Crohn‘s Disease”) OR (“Colitis” OR “Hemorrhagic Colitis” OR “Ulcerative Colitis”).

The initial evaluation and selection of scientific documents found in the databases were carried out independently by two researchers. For this process, the online software Rayyan was used, a tool that facilitates the screening and selection of scientific articles^10^.

The articles found in the databases were exported to the Rayyan interface in Research Information Systems (RIS) file format. Subsequently, a request was made for duplicate analysis, as this software allows for the automatic identification of duplicate studies and their subsequent exclusion, keeping only one version of each article.

After excluding the repeated articles, the theme and type of study were analyzed (phase 1) by reading the titles and abstracts of the articles. Then, the eligibility of the articles was evaluated by reading the complete articles (phase 2). Discrepancies in the study selection process were resolved by a third reviewer.

### Data extraction, analysis, and synthesis

Data extraction was performed using a form including: author names; impact factor (JCR 2020); year of publication; country; objectives; IBD studied; methodological design; sample size (if applicable); data collection period; main results; study limitations; conclusion and implications for clinical practice; level of methodological evidence.

The classification according to the level of evidence was based on the hierarchy and design of the studies. This method classifies studies into 7 levels, from I to VII, as described in Table 1. In this review, we consider levels I to III as strong, IV to VI as moderate, and VII as weak. Regarding the data synthesis, it was summarized descriptively and tabulated in the results section. The table contains (a) authorship/year; (b) country of the principal author; (c) objective; (d) clinical applicability; (e) methodological design; (f) level of evidence; and (g) JCR. Table 1 represents the level of evidence and study design in question.

**TABLE 1:** Level of evidence and study design.

Level of evidence	Study design
I	Systematic reviews or meta-analyses of randomized clinical trials.
II	Well-designed randomized controlled clinical trial.
III	Well-designed, non-randomized controlled clinical trial.
IV	Cohort study, case-control study, well-designed cross-sectional study.
V	Systematic review of qualitative and descriptive studies.
VI	Unique descriptive or qualitative study.
VII	Expert opinion and/or expert report.

Source: Author‘s own work (2024).

## RESULTS

**FIGURE 1:**
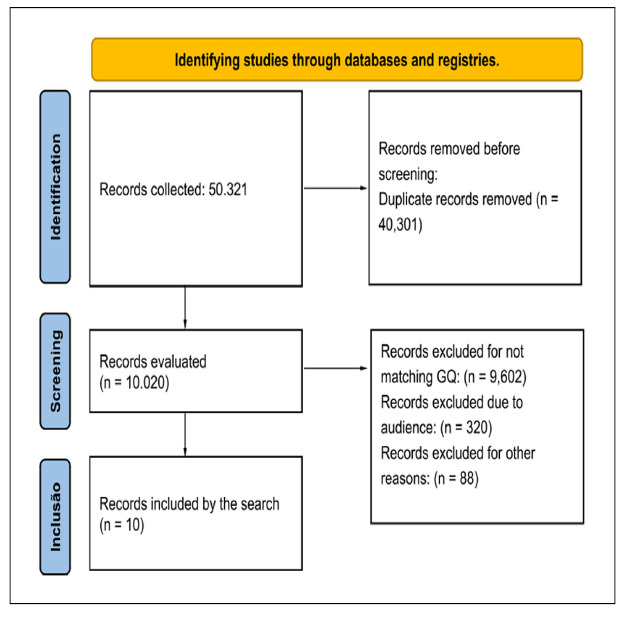
PRISMA-ScR Flowchart. Source: Author‘s own (2025). Adapted from Peters et al., (2021)^9^.

In the initial stage of the search, 50,321 articles were identified in the selected databases. After excluding 40,301 duplicate publications, 10,020 studies remained for the screening stage. Reading the titles resulted in the exclusion of those that were not related to the guiding question, leaving 10 articles that fully met the inclusion criteria and comprised this review. Figure 1 presents the flowchart with the stages of identification, screening, eligibility, and inclusion of the articles.

The included studies were published between 2000 and 2025, with the highest concentration from 2023 onwards, corresponding to 4 articles (40%), which highlights the growing scientific interest in investigating the effects of glucagon-like peptides (GLP-1 and GLP-2) in IBD.

Regarding the methodological design, a predominance of preclinical experimental studies (N=4; 40%) and retrospective cohort studies (N=3; 30%) was observed. In addition, integrative reviews (N=2; 20%) and a randomized clinical trial (N=1; 10%) were identified.

Regarding the type of disease addressed, 4 articles (40%) specifically investigated Crohn‘s Disease, 3 (30%) focused on Ulcerative Colitis, while 3 (30%) included both conditions. As for the object of study, 5 articles (50%) analyzed GLP-1^4^ ^,^ ^11^ ^-^ ^14^, while 5 articles (50%) focused on GLP-2^8^ ^,^ ^15^ ^-^ ^18^. No study investigated both peptides together.

In general, the findings suggest that GLP-1 has a relevant anti-inflammatory effect, especially in ulcerative colitis models^4^ ^,^ ^11^ ^-^ ^14^, while GLP-2 shows probable therapeutic potential, mainly in Crohn‘s Disease and conditions associated with intestinal failure^8^ ^,^ ^15^. Despite this, the studies are still heterogeneous in terms of design, population, and therapeutic regimen, limiting direct comparison between the results.

The main findings indicate that GLP-1 has an anti-inflammatory effect by suppressing pro-inflammatory cytokines such as IL-1β, IL-6, and TNF-α, improving intestinal barrier integrity, reducing the severity and activity of IBD, and positively modulating the microbiota^4^ ^,^ ^11^ ^-^ ^14^. In turn, the studies selected for this review demonstrate that GLP-2 tends to stimulate the proliferation of intestinal epithelial cells, reducing permeability, and favoring mucosal regeneration, thus modifying the bacterial flora^8^ ^,^ ^15^ ^-^ ^18^. Table 2 below summarizes the characteristics of the included studies, emphasizing the population, follow-up time, type and dose of GLP studied, and type of IBD studied.

**TABLE 2:** Synoptic table containing the characteristics of the included studies.

Citation	Title	General study data
Liu et al., 2025^11^	Dual activation of GCGR/GLP1R signaling ameliorates intestinal fibrosis *via* metabolic regulation of histone H3K9 lactylation in epithelial cells	**Study type:** Preclinical experimental study
		**Population:** Not described
		**Follow-up time:** 14 days
		**Type and dose of GLP studied**: GLP-1, the dose was 0.5 mg/kg administered subcutaneously (SC)
		**Type of IBD studied**: Crohn’s disease
Wang, Zhang, Zhang 2023^12^	The alleviating effect and mechanism of GLP-1 on ulcerative colitis	**Study type**: Preclinical experimental study
		**Population**: 48 mice
		Follow-up time: 7 days
		Type and dose of GLP studied: GLP-1, dose 0.2 mg/kg intraperitoneally (IP)
		**Type of IBD studied:** Ulcerative colitis
Zatorski et al., 2019^8^	Role of glucagon-like peptides in inflammatory bowel diseases-current knowledge and future perspectives	**Study type**: Integrative review
		**Population**: Not described
		**Follow-up time**: Not described
		**Type and dose of GLP studied:** GLP-1 and GLP-2, varying doses
		**Type of IBD studied:** Crohn’s disease and ulcerative colitis.
El-Jamal et al., 2014^15^	Glugacon-like peptide-2: broad receptor expression, limited therapeutic effect on intestinal inflammation and novel role in liver regeneration	**Study type**: Preclinical experimental study
		**Population:** Not described
		**Follow-up time**: 10 days
		**Type and dose of GLP studied:** GLP-2, the dose was 750 ng twice daily and 350 ng twice daily subcutaneously.
		**Type of IBD studied:** Ulcerative colitis
Villumsen et al., 2021^4^	GLP-1 based therapies and disease course of inflammatory bowel disease	**Study type**: Retrospective cohort study
		**Population**: 3751 participants
		**Follow-up time**: Not described
		**Type and dose of GLP studied:** GLP-1, dose not described
		**Type of IBD studied**: Crohn’s disease and ulcerative colitis.
Pizzoferrato et al., 2022^16^	Glucagon-like peptide-2 analogues for Crohn’s disease patients with short bowel syndrome and intestinal failure	**Study type:** Integrative review
		**Population:** Not described
		**Follow-up time:** Not described
		**Type and dose of GLP studied**: GLP-2, the dose was 0.05 mg/kg/day and 0.10 mg/kg/day, via daily subcutaneous injection
		**Type of IBD studied**: Crohn’s disease
Buchman et al,. 2010^17^	Teduglutide, a novel mucosally active analog of glucagon-like peptide-2 (GLP-2) for the treatment of moderate to severe Crohn’s disease	**Study type**: Randomized controlled clinical trial
		**Population:** 71 participants
		**Follow-up time**: 8 weeks
		**Type and dose of GLP studied:** GLP-2, at a dose of 0.05, 0.10, or 0.20 mg/kg/day
		**Type of IBD studied:** Crohn’s disease
Xiao et al,. 2000^18^	Circulating levels of glucagon-like peptide-2 in human subjects with inflammatory bowel disease	**Study type**: Cross-sectional observational study
		**Population**: 546
		**Follow-up time**: Not described
		**Type and dose of GLP studied:** GLP-2, dose not described
		**Type of IBD studied**: Crohn’s disease and Ulcerative Colitis
St-Pierre et al., 2024^13^	Efficacy and Safety of GLP-1 Agonists on Metabolic Parameters in Non-diabetic Patients with Inflammatory Bowel Disease	**Study type:** Retrospective cohort study
		**Population:** 36 patients
		**Follow-up time:** 3 years
		**Type and dose of GLP studied:** GLP-1, dose not described
		**Type of IBD studied:** Crohn’s disease and ulcerative colitis
Belinchon et al., 2025^14^	Effectiveness and safety of a GLP-1 agonist in obese patients with inflammatory bowel disease	**Study type:** Retrospective cohort study
		**Population:** 16 patients
		**Follow-up time:** 6 months
		**Type and dose of GLP studied:** GLP-1, at a dose of 1 mg semglutide and 3 mg liraglutide
		**Type of IBD studied:** Crohn’s disease and ulcerative colitis

SC: subcutaneous. IP: intraperitoneal.

Most of the available evidence was classified as moderate level (N=6; 60.0%), while studies rated as strong evidence accounted for a smaller proportion of the analyzed sample (N=3; 30.0%). One study was classified as weak evidence (N=1; 10.0%). This distribution indicates that, although relevant findings regarding the use of GLP-1 and GLP-2 in inflammatory bowel diseases exist, the body of evidence is predominantly composed of observational and cohort studies, many of them retrospective, as well as limited descriptive or opinion-based evidence. This scenario highlights the need for randomized, prospective, and methodologically robust clinical trials to support more consistent clinical recommendations. Table 3 presents a synthesis of the main clinical recommendations and the corresponding levels of evidence of the included studies. 

**TABLE 3:** Level of evidence and main clinical findings of the included studies.

Citation	Level of evidence	Main clinical findings
Liu et al., 2025^11^	VI - Weak	Treatment with the peptide 1907B (a dual agonist of GCGR/GLP1R) restores signaling at the receptors of these enzymes; reduces glycolysis and lactate production in epithelial cells; decreases H3K9 lactylation, inhibiting the activation of pro-fibrotic genes; and reduces intestinal fibrosis histologically.
Wang, Zhang, Zhang 2023^12^	III - Strong	Mice treated with GLP-1 analogs showed improvement in ulcerative colitis at the histological level; in these tissues, they obtained a reduction in the expression of inflammatory cytokines, such as IL-1β, IL-6 and TNF-α; modulation of the intestinal microbiota favoring the presence of beneficial microorganisms.
Zatorski et al., 2019^8^	V - Moderate	GLP-1 has suppressive effects on inflammatory cytokines in intestinal tissues, improves the intestinal barrier function by aiding in the integrity of the epithelium, assists in the remission of IBD, and reduces the severity and activity of IBD. GLP-2 stimulates the proliferation of intestinal epithelial cells and reduces permeability, stimulates the regeneration of the intestinal mucosa, and alters the intestinal bacterial flora.
El-Jamal et al., 2014^15^	III - Strong	The expression of GLP-2 receptors: was found in extraintestinal media, mainly in the liver; its expression was reduced by up to 60% in tissues extensively affected by inflammation. The use of GLP-2 analogs aided in liver regeneration in mice after partial hepatectomy, with histological evidence.
Villumsen et al., 2021^4^	IV - Moderate	The use of GLP-1 analogs did not increase IBD activity in participants, with typical gastrointestinal symptoms occurring, but without flare-ups of the underlying disease. This medication is linked to a lower risk of hospitalizations and surgeries, in addition to enabling the control of inflammation, avoiding the escalation of immunosuppressive drugs. GLP-1 reduces the expression of inflammatory cytokines (TNF-α, IL-6, IL-1β), aids in the integrity of the intestinal mucosa, and modulates the intestinal microbiota with an increase in beneficial microorganisms.
Pizzoferrato et al., 2022^16^	V - Moderate	The use of teduglutide in patients with Crohn’s disease after multiple surgical procedures and nutritional deficiencies has proven viable in reducing or even eliminating the need for parenteral nutrition in these patients. It presents only mild to moderate side effects, such as nausea or mild abdominal pain.
Buchman et al,. 2010^17^	II - Strong	Teduglutide is potentially effective in inducing remission and mucosal healing in patients with moderate to severe active Crohn’s disease.
Xiao et al,. 2000^18^	IV - Moderate	Serum GLP-2 levels were found in patients with IBD that were higher than in control patients, being 229% higher in ulcerative colitis and 317% higher in Crohn’s disease. Similarly, the active form of GLP-2 was higher in IBD patients, and the DPP-IV enzyme that degrades GLP-2 was lower in these patients. Therefore, the elevated GLP-2 levels in these patients are linked to an adaptive response to intestinal inflammation.
St-Pierre et al., 2024^13^	IV - Moderate	GLP-1 analogs, such as semaglutide and tirrizepatide, can effectively reduce weight and BMI in non-diabetic patients with IBD, with manageable side effects.
Belinchon et al., 2025^14^	IV - Moderate	The use of GLP-1 agonists was effective in reducing weight by 6.2% in 6 months, showing a good safety profile in IBD. The most frequent adverse event was nausea. Commonly, GLP-1 agonists cause mild gastrointestinal symptoms such as nausea, vomiting, and diarrhea. The improved course of IBD may be explained by the reduction of intestinal inflammation through the interaction between GLP-1 agonists and intestinal intraepithelial lymphocytes. Treatment with GLP-1-SSM reduced the expression of the pro-inflammatory cytokine IL-1β, increased goblet cells, and preserved the intestinal epithelial architecture in a colitis model.

IBD: inflammatory bowel disease. UC: ulcerative colitis. CD: crohn‘s disease.

## DISCUSSION

### Metabolic disorders and IBD

**FIGURE 2:**
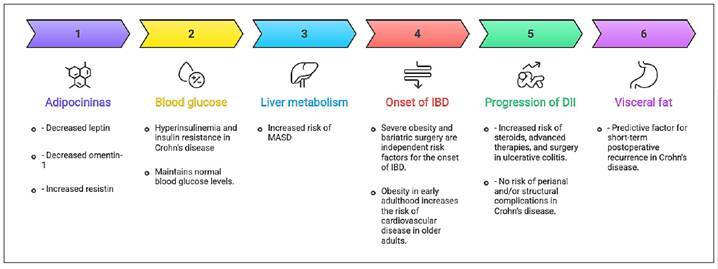
Metabolic interactions between inflammatory bowel disease and obesity. CD: Crohn‘s disease. UC: Ulcerative Colitis. MASLD: Metabolic Dysfunction-Associated Steatotic Liver Disease. IBD: Inflammatory Bowel Disease. Source: Adapted from Carrillo-Palau et al., (2021)^22^; Nie (2015)^23^; Cañete et al., (2025)^24^; Desai et al., (2025b)^25^; Youn et al., (2024)^26^. Figure created using NapkinIA software.

The prevalence of metabolic disorders, such as type 2 diabetes mellitus (DM2) and obesity, has been progressively increasing among individuals with IBD. It is estimated that between 15% and 40% of IBD patients are obese, while another 20% to 40% are overweight^19^. IBD and metabolism are closely interconnected (Figure 2), and several lines of evidence indicate that adipokine levels are frequently altered in these patients^20^ ^,^ ^21^.

Leptin levels tend to be reduced during the active phase of IBD, possibly as a result of depletion following transient overproduction associated with TNF-α hyperactivity. In contrast, resistin levels are usually elevated, correlating with activation of the NF-κB pathway and increased secretion of pro-inflammatory cytokines such as TNF-α, IL-6, and IL-1β^27^. Even in the presence of normoglycemia, elevated serum resistin levels have been associated with hyperinsulinemia in active IBD^28^, suggesting that systemic inflammation may contribute to the development of insulin resistance (IR), independently of baseline glycemia.

Furthermore, patients with IBD have an increased risk of insulin resistance, particularly those with Crohn’s disease^29^ ^,^ ^30^. However, recent studies do not consistently confirm this association, indicating that the increased risk of IR may be more strongly related to the concomitant presence of metabolic dysfunction-associated steatotic liver disease (MASLD)^22^. This finding suggests that chronic inflammation in IBD may act as a predisposing factor for metabolic dysfunction, often in interaction with pre-existing metabolic comorbidities.

On the other hand, obesity and insulin resistance themselves appear to act as risk factors for the development and poorer prognosis of IBD. Omentin-1-an adipokine with anti-inflammatory properties-has reduced levels in patients with IBD^23^, highlighting the interdependence between visceral adipose tissue and intestinal inflammation. Severe obesity and bariatric surgery have been identified as independent risk factors for the onset of IBD^24^. Additionally, obesity in young adults is associated with a significantly increased risk of late-onset Crohn’s disease^31^. In patients with Ulcerative Colitis, a propensity score-matched cohort study demonstrated that obesity is a determinant of greater corticosteroid use, need for therapeutic escalation, and increased risk of colectomy^32^. Conversely, no increased risk of perianal or stricturing complications was observed in individuals with Crohn’s disease^33^.

Thus, current evidence suggests a bidirectional interaction: chronic inflammation in IBD may promote metabolic alterations, including insulin resistance and hyperinsulinemia, while obesity and metabolic dysfunction may contribute both to the development and to greater severity and therapeutic refractoriness of IBD.

### Role of GLP-1 agonist in the regulation of inflammation in IBD

The therapeutic arsenal for the treatment of IBD has expanded significantly in recent decades. Among the established mechanisms, the following stand out: anti-TNF agents, anti-IL12/IL23 agents, anti-integrins, selective IL-23 inhibitors, and small molecules modulating the JAK pathway^17^ ^,^ ^34^ ^,^ ^35^.

Initially used in the management of type 2 diabetes mellitus and obesity, GLP-1 receptor agonists exhibit intestinal anti-inflammatory properties, associated with improved glycemic control, weight reduction, modulation of gastric emptying, and decreased food intake, characteristics that make them potential modulators of intestinal inflammation^8^ ^,^ ^13^ ^,^ ^17^.

Preclinical and observational evidence indicates that GLP-1 acts in the modulation of intestinal inflammation, interfering with immunological pathways, the integrity of the epithelial barrier, and the composition of the microbiota. These mechanisms suggest potential benefit in patients with overweight, obesity, or metabolic syndrome, conditions that aggravate the inflammatory activity of IBD^4^.

Experimental models of induced colitis in animals have shown that GLP-1 agonists reduce the expression of pro-inflammatory cytokines, such as TNF-α, IL-1β, and IL-6, and increase the release of IL-10, promoting reinforcement of the epithelial barrier, tissue regeneration, and improved intestinal permeability. These effects appear to result from the activation of GLP-1 receptors in epithelial and immune cells, with a beneficial impact independent of glycemic control. GLP-1 also modulates intestinal transit and motility, influencing antigenic contact and microbial composition-central factors in the pathophysiology of IBD^12^.

In addition to immunological modulation, GLPs contribute to epithelial repair and regulate the differentiation of T cells, macrophages, and dendritic cells, reducing the release of inflammatory cytokines. Unlike biological therapies, which act with a systemic immunosuppressive effect and may favor opportunistic infections, GLP-1 agonists do not present this profile, constituting a promising and potentially safer alternative for the treatment of IBD^8^ ^,^ ^14^ ^,^ ^17^.

In obese patients with inflammatory bowel diseases, some observational studies have demonstrated favorable outcomes in weight control, without evidence of worsening disease activity during the use of GLP-1 agonists. In a sample of 16 obese individuals (BMI ≥30 kg/m²), a mean reduction of 6.2% in body weight was observed over six months of follow-up, with no increase in clinical outcomes suggestive of IBD exacerbation. However, these studies did not systematically assess objective parameters of intestinal inflammatory activity, such as laboratory markers, endoscopic findings, or validated clinical indices, which precludes conclusions regarding a potential direct or indirect anti-inflammatory effect of GLP-1 agonists in this context. Therefore, the findings should be interpreted as indicative of short-term metabolic and clinical safety rather than evidence of inflammatory modulation of IBD. Prospective, controlled studies with specific inflammatory endpoints remain necessary to clarify this potential effect^14^ ^,^ ^17^.

Initially, the main concern regarding the use of GLP-1 receptor agonists in patients with IBD and metabolic comorbidities was the potential exacerbation of intestinal inflammation, given the well-known gastrointestinal adverse effects of this pharmacological class-such as nausea, vomiting, and altered bowel habits. Therefore, early studies primarily aimed to assess the safety of these agents in this patient profile. In humans, although data remain limited, the available evidence indicates that the use of GLP-1 agonists in patients with IBD associated with obesity or diabetes does not increase the risk of disease exacerbation, hospitalization, or need for intestinal surgery^4^.

Beyond a favorable safety profile, significant reductions in inflammatory markers, improvement in gastrointestinal symptoms, and enhancement of metabolic parameters have been observed, suggesting a possible indirect benefit on intestinal inflammatory activity^17^ ^,^ ^36^. In a cohort published in 2024, patients with Crohn’s Disease and Ulcerative Colitis associated with obesity who were treated with semaglutide showed a lower risk of exacerbation and better metabolic control, without an increase in severe gastrointestinal events^25^. These findings support the hypothesis that weight loss, improvement in insulin resistance, and reduction of pro-inflammatory mediators may contribute to modulation of intestinal inflammation.

However, randomized controlled clinical trials are still required to definitively confirm these results and establish formal recommendations for use in this population. To date, only one phase III clinical trial is ongoing, aiming to evaluate the combination of mirikizumab, a selective IL-23 inhibitor, with tirzepatide, a dual GLP-1 and GIP receptor agonist, in order to compare the efficacy and safety of combination therapy versus mirikizumab monotherapy. No results are currently available, but the study underscores the translational potential of targeting these pathways^37^.

Despite the advances, the available evidence is predominantly observational and based on small samples, which limits the strength of the conclusions. Controlled clinical trials evaluating the impact of GLP-1 agonists on the clinical and endoscopic activity of IBD are lacking. The pharmacological heterogeneity among agents in this class, including differences in receptor affinity and pharmacokinetic profiles, also requires individualized analysis. Furthermore, most studies were conducted in patients in remission, and data in populations with active disease are needed^4^ ^,^ ^8^ ^,^ ^12^ ^,^ ^17^.

The therapeutic role of GLP-1 agonists in IBD remains under investigation. It is plausible that, in the future, these agents will become adjuvant options in the management of intestinal inflammation, especially in patients with obesity, diabetes, or insulin resistance. Until further clinical evidence is established, the use of these drugs should be carefully evaluated, considering their safety profile and their effects on gastrointestinal motility and function^15^ ^,^ ^36^ ^,^ ^38^.

### Role of GLP-2 agonist in regulating inflammation in IBD

GLP-2 is an endocrine peptide derived from the post-translational processing of proglucagon in the intestine. Structurally related to the proglucagon-derived peptide, GLP-1, GLP-2 shares the same precursor and is produced and secreted by enteroendocrine L cells of the small and large intestine, in a nutrient-dependent manner^15^.

The administration of teduglutide, a GLP-2 analog, has demonstrated significant effects in experimental models of colitis, highlighting its therapeutic potential in modulating the intestinal response to inflammation. In mice with DSS-induced colitis, treatment with teduglutide promoted a significant increase in the weight and length of the small intestine and colon, associated with improved histological morphology and stimulation of crypt cell proliferation, compared to untreated animals^39^.

Comparative studies have indicated that the efficacy of teduglutide is equivalent to that observed with sulfasalazine and corticosteroids in attenuating intestinal inflammatory processes^40^. In an HLA-B27 colitis model, daily administration of 50 μg/kg of teduglutide significantly reduced histological damage in the small and large intestine, decreased the expression of inflammatory cytokines, including IFN-γ and TNF-α, and promoted improved fecal consistency compared to the control group^41^ ^,^ ^42^.

Further investigations have demonstrated that the administration of GLP-2 in TNBS- and DSS-induced colitis models resulted in increased crypt depth and villus height, concomitant with a reduction in pro-inflammatory markers such as IL-1β, IFN-γ, and TNF-α^41^ ^,^ ^42^.

Furthermore, there is a reduction in crypt proliferation and apoptosis, attenuation of pro-inflammatory cytokine production, and an increase in IL-4, accompanied by a decrease in the population of CD4(+) T cells. The anti-inflammatory effects of GLP-2 occur independently of IL-10, modulating the intestinal mucosal response by reducing TNF-α produced by lamina propria macrophages and increasing insulin-like growth factor 1 (IGF-1)^43^ ^-^ ^44^.

Considering the short half-life of native GLP-2 in human circulation, recent research has focused on the development of more stable analogs capable of prolonging the intestinotropic action of this molecule. Teduglutide, whose half-life is modestly extended to 3-5 hours, has shown promising results, but the need for drugs with a longer duration of action has driven the creation of new GLP-2 derivatives^44^. Yang et al. (2018)^45^ investigated peptide-10, an analogue with a half-life approximately ten times longer than that of native GLP-2, which demonstrated superior efficacy and more pronounced intestrotrophic effects in a DSS-induced colitis model compared to teduglutide.

**FIGURE 3:**
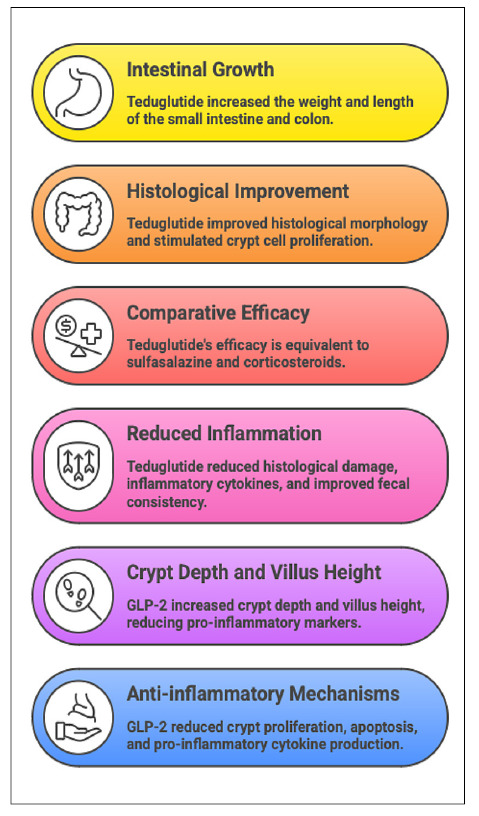
GLP-2 effects in colitis model. Source: Author‘s own (2026).Figure created using NapkinIA software.

The expression of the glucagon-like peptide-2 receptor (GLP-2R) was evaluated in multiple cell models, murine tissues, and human samples, including intestinal cell lines (Caco-2, HT-29, 5F7), colonic myofibroblasts (CCD-18Co), primary hepatocytes and immortalized cell lines, intestinal endocrine cells, and immune system cells^15^. Figure 3 below summarizes these findings.

In human samples, GLP-2R expression was higher in the colon compared to the ileum in healthy individuals and patients with IBD. Intestinal inflammation significantly reduced GLP-2R expression in murine models of TNBS- and DSS-induced colitis, as well as in inflamed and non-inflamed tissues of IBD patients. GLP-2 agonist intervention trials in murine colitis showed an intestrotrophic effect, with increased colonic length, but did not significantly reduce the severity of inflammation, as assessed macroscopically and by IL-1β levels. These findings indicate that, although GLP-2R is widely expressed in specific tissues, its activation has limited physiological effects outside the distal intestine, restricting its therapeutic potential in modulating intestinal inflammation^15^ ^-^ ^18^.

Glepaglutide, a GLP-2 analog, has demonstrated in experimental models that activation of the GLP-2R receptor in the gastrointestinal tract promotes a reduction in inflammatory severity and improved intestinal recovery, both in early and late treatments. This intestinal-inflammatory mechanism suggests that GLP-2 and its analogs have great therapeutic potential to induce mucosal healing and maintain remission in IBD, overcoming the limitations of traditional therapies focused on the immune system^18^ ^,^ ^46^.

In humans, Crohn‘s disease patients undergoing intestinal resection have reduced levels of total GLP-2 due to the loss of enteroendocrine-producing cells, especially in the distal ileum. Furthermore, the activity of DPP IV, responsible for GLP-2 degradation, is decreased in IBD patients, which may increase the proportion of circulating active GLP-2. This regulation of GLP-2 homeostasis, through production and degradation, is fundamental to the physiological response to intestinal inflammation and injury, highlighting GLP-2 as a promising target for promoting intestinal repair and regeneration in patients with IBD^18^ ^,^ ^44^ ^,^ ^46^.

The use of teduglutide in patients with Crohn‘s disease who have undergone multiple surgical interventions and present nutritional deficiencies has proven viable and effective in reducing, and in many cases even eliminating, the need for parenteral nutrition. Studies demonstrate that, in a significant proportion of these patients, teduglutide promotes a significant reduction in the volume of parenteral support and allows the transition to greater enteral autonomy, resulting in improved quality of life. The observed side effects were generally mild to moderate, including nausea and mild abdominal pain, which reinforces the acceptable safety profile of the therapy^16^ ^,^ ^17^.

These clinical results make teduglutide a promising option for the management of short bowel syndrome associated with Crohn’s disease, especially in patients with chronic nutritional dependence after multiple intestinal resections, where current therapeutic alternatives are limited. It is important to highlight that, despite the advances, long-term studies are still needed to broaden the understanding of its continued efficacy, safety profile, and impact on the quality of life of these patients, consolidating its use more widely^16^ ^,^ ^17^.

This scoping review has limitations that should be considered when interpreting its findings. A potential publication bias is evident, as despite the large number of records initially identified across databases, only a limited number of studies met the eligibility criteria, which may overestimate positive effects and underrepresent neutral or negative outcomes. Moreover, most of the available human evidence derives from patients in clinical remission or with stable disease, thereby restricting the extrapolation of these findings to individuals with moderate to severe inflammatory activity, in whom the effects of GLP analogues may differ substantially.

From a translational perspective, the findings suggest distinct therapeutic prospects for GLP-1 and GLP-2 analogues in inflammatory bowel disease. GLP-1 receptor agonists appear particularly promising in patients with inflammatory bowel disease associated with obesity or type 2 diabetes mellitus, as they may simultaneously target inflammatory pathways and metabolic dysfunction. In contrast, teduglutide, a GLP-2 analogue currently used in short bowel syndrome, suggests a potential role focused on mucosal repair and restoration of intestinal barrier integrity, independent of metabolic comorbidities, and may represent a future strategy in the management of active disease. However, intraclass heterogeneity among these agents-including differences in pharmacokinetics, pharmacodynamics, receptor affinity, and safety profiles, which remain insufficiently explored-limits direct comparisons and hinders the formulation of robust clinical recommendations.

## CONCLUSION

The currently available evidence suggests that glucagon-like peptides (GLP-1 and GLP-2) exert biologically relevant effects on the gastrointestinal tract, with potential implications for mechanisms involved in inflammatory bowel diseases. Preclinical and observational studies indicate that GLP-1 may modulate inflammatory responses, suppress pro-inflammatory cytokines, contribute to intestinal barrier integrity, and influence the gut microbiota, particularly in models of ulcerative colitis. GLP-2, in turn, has well-established intestinotrophic effects, promoting mucosal regeneration, reducing intestinal permeability, and improving nutrient absorption, with more consistent clinical applicability in specific contexts, such as intestinal failure associated with Crohn’s disease.

However, it is essential to emphasize that most of the available evidence derives from preclinical or observational studies, with a paucity of randomized clinical trials targeting IBD-specific outcomes. Therefore, although GLPs represent a biologically plausible and increasingly relevant field of investigation, the current evidence does not support their indication as established therapies for IBD. Their clinical use should be interpreted with caution and remain restricted to approved indications or specific clinical contexts until robust, controlled clinical trials focused on inflammatory bowel disease activity are conducted. Future research is required to elucidate the efficacy, safety, clinical applicability, and potential intraclass differences of these agents in the management of IBD.

## Data Availability

The research data are presented within the article itself (available in Results section).
